# COVID 19 pandemic, status of clinical trials in Africa on May 2020: need to reinforce

**DOI:** 10.11604/pamj.supp.2020.35.2.24349

**Published:** 2020-06-22

**Authors:** Jan René Nkeck, Aude Laetitia Ndoadoumgue, Mazou Ngou Temgoua

**Affiliations:** 1 Faculty of Medicine and Biomedical Sciences, University of Yaoundé I, Cameroon; 2 Ecole des Hautes Etudes en Santé Publique, Rennes, France

**Keywords:** COVID-19, clinical trials, Africa

## To the Editors of the Panafrican Medical Journal

Coronavirus disease 2019 (COVID-19) is an unprecedented global crisis caused by a new strain of coronavirus called SARS-Cov 2 (Severe Acute Respiratory Coronavirus 2). It was first identified in the city of Wuhan, capital of Hubei Province in China and has rapidly spread across the world. On March 11, 2020 the disease was recognized as pandemic by the World Health Organization (WHO) [1]. Between 31 December 2019 to 05 May 2020, 3 544 222 cases of COVID-19 have been reported, including 250 977 deaths [2]. According to the Center for Disease Control (CDC), Africa has the 4th highest number of cases with 47 124 cases behind America, Europe and Asia on the 5th May 2020. In this continent, the most affected countries are South Africa (7,220), Egypt (6,813), Morocco (5,043), Algeria (4,648) and Nigeria (2 802) [2]. The virus primarily affects the lower respiratory tract, manifesting as acute pneumonia in humans and responsible for severe complications in patients with various comorbidities such as old age, pregnancy, cancer, chronic respiratory and cardiovascular diseases [3]. So far, real-time RT-PCR assays with full genome sequencing and phylogenetic analysis on fluid from bronchoalveolar lavage remain the gold standard for the diagnosis of SARS-CoV-2 infection [4]. Considering the high burden of the disease, therapeutic options in response to the COVID-19 outbreak are urgently needed. However, given that the development of new drugs in the context of a pandemic is generally complex and requires ample time, the most cost-effective strategy is to repurpose existing drugs [5]. Selected drugs which are currently being repurposed include agents with apparent in vitro activity against SARS-CoV and MERS-CoV (Hydroxychloroquine, Lopinavir/Ritonavir and other antiretrovirals) and adjunctive therapies (corticosteroids, anticytokine or immunomodulatory agents, and immunoglobulin therapy) [6]. These temporary therapeutic solutions will be revised with the availability of new validated drugs. Therefore, in light of the spreading of the disease in Africa it is crucial to determinate the continent´s implication in clinical trials.

**Figure 1:**
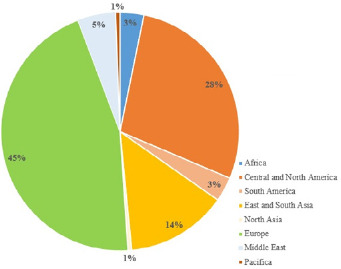
Percentage of clinical trials on COVID 19 per region in the world on the 5th May 2020

In order to assess the implication of African countries in clinical trials it was imperative to conduct this study. We researched as of May 5, 2020 the ClinicalTrials.gov website to identify registered clinical trials on Coronavirus 2019 [7]. We´ve used the following acronyms with no restriction: “COVID” OR “COVID-19” OR “SARS-CoV-2” OR “2019-nCoV” OR “2019 novel coronavirus” OR “Coronavirus 2019” OR “severe acute respiratory syndrome coronavirus 2” OR “Wuhan coronavirus”. We identified 1209 clinical trials, of which 1002 had information concerning the host country. Among the clinical trials registered to date, there were 722 interventional studies, 549 trials with recruitment underway, 44 clinical trials already completed and one terminated. The majority were hosted by a European country (449), followed by North and Central American countries (279). Countries in East and South Asia, where the pandemic began, have 136 clinical trials to date. Africa has 32 clinical trials after the Middle East (51) and South America (33) (Figure 1). In Africa, the participation in clinical trials is disproportionate. Egypt has the largest number of trials (24) with 7 in progress, followed by Tunisia (4) and South Africa (2). Apart from Egypt, most clinical trials in other African countries are multinational. More than two thirds (23/32) of the clinical trials registered in Africa are of the interventional type. Funding for trials conducted and/or planned in Africa is largely from individual funds, academic funding, and organisations.

**Figure 2:**
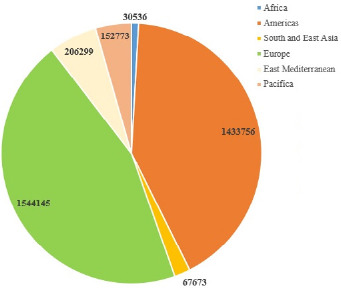
Prevalence COVID 19 in the World on the 5th May 2020

Although the pandemic started late in Africa, the current number of cases exceeds 30,000, representing almost half of the total cases detected in East and South Asia to date (Figure 2). The experts predicted that African population could be more at risk of developing COVID-19 with more severe cases compared to other populations. In fact, transmission may be favoured by low socio-economic status, promiscuity, poor hygiene, coupled with the high burden of chronic communicable (HIV) and non-communicable (hypertension, diabetes) diseases [8]. There is currently little data to understand Africa’s low involvement in COVID 19 clinical trials, however, one can cite the heavy reliance on traditional medicine, with the application by some governments of treatment protocols that have not been validated through clinical trials [9]; there is also public mistrust due to the controversy over vaccine clinical trials in Africa [10]. Nevertheless, this pandemic calls for a global response and the need to respect the canons of scientific research aimed at proving the effectiveness of a treatment. This is a wake-up call to the African scientific community to be fully involved in clinical research on COVID 19 in order to share its therapeutic potential with nations and regions at large. Africa´s involvement in clinical trials despite the rapid spread of COVID-19 in this continent is still quite underwhelming. Like the rest of the world, Africa was unprepared for this unprecedented pandemic, but given its spread throughout the continent, it is urgent to reinforce participation in clinical trials so as to find appropriate drugs to curb the burden of the disease.

## Competing interests

The authors declare no competing interests.
